# Magnitude of Neural Tube Defects and Associated Risk Factors at Three Teaching Hospitals in Addis Ababa, Ethiopia

**DOI:** 10.1155/2018/4829023

**Published:** 2018-03-11

**Authors:** Abel Gedefaw, Sisay Teklu, Birkneh Tilahun Tadesse

**Affiliations:** ^1^Department of Gynecology and Obstetrics, College of Medicine and Health Sciences, Hawassa University, Hawassa, Ethiopia; ^2^Department of Gynecology and Obstetrics, College of Medicine and Health Sciences, Addis Ababa University, Addis Ababa, Ethiopia; ^3^Department of Pediatrics and Child Health, College of Medicine and Health Sciences, Hawassa University, Hawassa, Ethiopia

## Abstract

There is scarcity of data on prevalence of neural tube defects (NTDs) in lower-income countries. Local data are important to understand the real burden of the problem and explore risk factors to design and implement preventive approaches. This study aimed to determine prevalence and risk factors of NTDs. A hospital-based cross-sectional and unmatched case-control study was conducted at three teaching hospitals of Addis Ababa University. NTDs were defined as cases of anencephaly, spina bifida, and encephalocele based on ICD-10 criteria. The prevalence of NTDs was calculated per 10,000 births for both birth and total prevalence. During seven months, we observed 55 cases of NTDs out of 8677 births after 28 weeks of gestation—birth prevalence of 63.4 per 10,000 births (95% confidence interval (CI), 51–77). A total of 115 cases were medically terminated after 12 weeks of gestation. Fifty-six of these terminations (48.7%) were due to NTDs. Thus, total prevalence of NTDs after 12 weeks' gestation is 126 per 10,000 births (95% CI, 100–150). Planned pregnancy (adjusted odds ratio (aOR), 0.47; 95% CI, 0.24–0.92), male sex (aOR, 0.56; 95% CI, 0.33–0.94), normal or underweight body mass index (aOR, 0.49; 95%, 0.29–0.95), and taking folic acid or multivitamins during first trimester (aOR, 0.47; 95%, 0.23–0.95) were protective of NTDs. However, annual cash family income less than $1,300 USD (aOR, 2.5; 95%, 1.2–5.5), $1,300–1,800 USD (aOR, 2.8; 95%, 1.3–5.8), and $1,801–2,700 USD (aOR, 2.6; 95%, 1.2–5.8) was found to be risk factors compared to income greater than $2,700 USD. The prevalence of NTDs was found to be high in this setting. Comprehensive preventive strategies focused on identified risk factors should be urgently established. More studies on prevention strategies, including folic acid supplementations, should be conducted in the setting.

## 1. Introduction

Neural tube defects (NTDs), a common group of central nervous system anomalies, comprise a major public health problem. It is estimated that approximately 300,000 babies are born each year with NTDs worldwide [1]. According to the World Health Organization (WHO) in 2010, an estimated 270,000 neonatal deaths globally were attributable to congenital anomalies, with NTDs being one of the most serious and most common of these anomalies [2, 3].

Both the birth and total prevalence of NTD have declined during the past three decades among high income countries [4–6]. Evidence suggests that the reduction in the birth prevalence of NTDs is largely due to termination of pregnancy after the introduction of routine serum alpha-fetoprotein measurements and advances in ultrasonography resolution for in utero early detection and termination of affected pregnancies [5, 6]. Additionally, improvements in folic acid supplementation have contributed to the reduction in total prevalence of NTDs [6]. For example, the incidence of myelomeningocele in Seattle, Washington, USA, was 5 per 10,000 births in 1981–1982, which then declined to 0.5 in 2001 [7]. The prevalence of NTDs in England and Wales was 38.0 per 10,000 live births in 1965, which steadily declined to 1.4 in 1997, a reduction of 96% [8]. Contrary to the developed world, prevalence of birth NTDs in developing countries is still high, with reported incidence as high as 130 per 10,000 births [9].

Data on the prevalence of NTDs are limited in lower-income countries despite the WHO resolution on birth defect surveillance [10]. A recent systematic review showed that many WHO member states (120/194) did not have any data on NTD prevalence [11]. Similarly, NTD prevalence in Ethiopia is not clearly known, and the country currently has no national preventive strategies. A retrospective chart review from Addis Ababa teaching hospitals revealed the overall prevalence of NTDs to be 6.1/1000 births [12]. The review concludes by recommending prospective studies to understand the real burden of NTDs.

Given the high prevalence of NTDs in the Ethiopian setting from retrospective data, it is imperative to investigate the burden and pattern of NTDs by prospectively following delivery outcomes. Moreover, to suggest plausible recommendations regarding preventive strategies against these disorders, risk factor assessment using a case-control design is important. In the current study, as there is no established preventive strategy for NTDs in Ethiopia [12, 13] and as the country is mostly a low income setting with low educational attainment and poor socioeconomic status, we hypothesize that the prevalence of NTDs in Ethiopia would be high and the risk factors associated with their occurrence are different from studies in high income settings. Hence, the current study sets out to investigate the prevalence of NTDs among pregnancy terminations and deliveries in major teaching hospitals in Addis Ababa. Additionally, the risk factors associated with NTDs are explored.

## 2. Methods and Materials

### 2.1. Study Area, Period, and Design

The study was conducted in Addis Ababa, Ethiopia's largest urban center and capital city, with 3.3 million inhabitants with male to female ratio of 0.91 in 2013 [14]. According to Ethiopian Demographic and Health Survey (EDHS) 2011, the coverage of antenatal care (ANC) services, birth assistance by skilled provider, and postnatal care in the city were 93.6%, 83.9%, and 47.7%, respectively [13].

The current study used two study designs, namely, a prospective cross-sectional and a hospital-based case-control study. A prospective registration of all birth outcomes was conducted between February 1, 2016, and August 30, 2016, at three major hospitals in Addis Ababa to understand the prevalence of NTDs. An institution-based, unmatched case-control study was conducted to identify the risk factors associated with NTDs. The three hospitals, Tikur Anbessa Specialized Hospital (TAH), Zewditu Memorial Hospital (ZMH), and Gandhi Memorial Hospital (GMH), are the major teaching hospitals for Addis Ababa University. They provide 24-hour obstetrics and gynecology care for the city. The total number of annual deliveries in the three hospitals is more than 15,000 with a Cesarean section (CS) rate of 30–40%. Most of the deliveries and evaluations are made by obstetrics and gynecology residents under the supervision of senior obstetrician and gynecologists.

Sample size of the study was calculated separately for both the prevalence and risk factors to NTDs. For the prevalence, considering the prevalence of NTDs to be similar to a retrospective study in Addis Ababa of 6% [12], and using a single proportion formula at 95% CI and 0.5% margin of error, a total of 8,663 pregnant mothers had to be enrolled to have 80% power in estimating the burden of NTDs in the setting. Based on this sample size, and considering the delivery rate at the hospitals, at least a seven-month duration of data collection was decided. A total of 8,677 deliveries were evaluated during the predefined data collection period. For assessing risk factors for NTDs, using Epi Info, Stat Calc, and considering 95% confidence level (CI), 80% power, control to case ratio of 2, minimum odd ratio (OR) of 2, and 10% nonresponse gave a minimum sample of 111 cases of NTDS and 222 controls.

### 2.2. Data Collection Process and Analysis

All intern doctors, residents, midwives, and clinical nurses were informed to report to the principal investigator whenever suspected cases of NTDs were delivered or medically terminated. Afterwards, data were collected by trained intern doctors and residents working at the selected hospitals in the postpartum or postabortal period and before discharge of the women. NTDs were defined as cases of anencephaly, spina bifida, and encephalocele (ICD-10) [15] among infants of any gestational age and medically terminated NTDs incompatible with life. Cases were ascertained by two senior obstetrics and gynecology residents by gross visual examination [16]. Radiologists used ultrasonography to perform prenatal diagnosis for termination of pregnancy, and senior obstetrics and gynecology residents confirmed diagnoses after expulsion by gross appearance of the abortus.

For the case-control design, to assess risk factors for NTDs, for each confirmed case of NTD, two mother-baby dyads with a normal delivery were selected systematically from that same day's delivery list of the same hospital. Cases with any ambiguity or multiple congenital anomalies were excluded from the study to avoid misclassification. Live birth or stillbirth was considered after 28 weeks of gestation or birth weight of 1,000 gm and greater. Elective termination of pregnancy for NTDs was made after 12 weeks of gestations and diagnosis via ultrasonography. NTDs cases were identified and ascertained within one week of birth or termination.

Data were collected by face-to-face interviews using a structured questionnaire adapted from the WHO birth defect surveillance tool [16]. Data were entered and analyzed using SPSS version 20 statistical package. The prevalence of NTDs calculated for both birth prevalence and total prevalence per 10,000 based on birth outcomes [live births (LBs), stillbirths (SBs), and terminated pregnancy]. Birth prevalence of NTDs is defined as number of cases of LBs or SBs with NTDs after 28 weeks of gestation (numerator) among a total number of LBs plus SBs during the study period. Total prevalence of NTDs is defined as number of NTD cases of LBs, SBs, and terminated pregnancy for fetal NTDs (numerator) among a total of LBs, SBs, and terminated cases beyond 12 weeks of gestation during the study period. The 95% CI for prevalence of NTDs was calculated using the binomial distribution as the number of cases in each category was greater than 30. Both bivariate and multivariate analyses were done to identify risk factors of NTDs. Out of the total independent variables, *p* values of less than 0.25 were taken for multivariate analysis. Stepwise backward multivariate logistic regression analysis was used to identify the best model with maximum predictive ability, which was used for final analysis. *p* value less than 0.05 was used as cut-off point for multivariate analysis.

In the current study, preconception was used to refer to the preceding three months before conception, while periconception was used to refer to the period including three months before conception (preconception) and the first three months after conception (first trimester). Preconception care is any type of care (health promotion, treatment, and supplementation) provided to a woman before conception. Drugs in this study means both prescribed pharmaceuticals and over the counter medications, not drugs used for recreations.

### 2.3. Ethical Consideration

Ethical clearance was obtained from the institutional review board (IRB) of College of Medicine and Health Sciences of Addis Ababa University (CMHS, AAU). A formal letter obtained from CHMS, AAU requesting permission to cascade the study was sent to each hospital's medical director. After the purpose of the study was described to the participating women, written consent was obtained from each one of them. In order to maintain confidentiality of any information provided by study subjects, the data collection procedure was anonymous. Participants with NTDs were counselled about the condition of their baby on the day following their delivery and the benefits of preconception folic acid supplementation to decrease future recurrence of NTDs.

## 3. Results

### 3.1. Sociodemographic Characteristics of the Participants

A majority of the cases (94.6%) and controls (94.1%) were from Addis Ababa. There was no difference between the mean age of cases (26.9; ±4.6) and controls (26.7; ±5.2) (*p* value; 0.193). Moreover, there was no difference between the cases and controls in terms of the participants' religion, marital status, educational status, family size, and occupation. However, there was a statistically significant difference in prepregnancy body mass index (BMI) (*p* value; 0.041) and family annual cash income (*p* value; 0.032) [Table 1].

### 3.2. Obstetric History of Participants

There was no difference between the cases and controls in terms of history of abortion (*p* value; 0.198), stillbirth (*p* value; 0.364), and congenital anomaly birth (*p* value; 0.820). There were three (2.7%) cases of congenital anomaly (all NTDs) in the cases, and seven (3.2%) cases of congenital anomaly (five NTDs and two congenital heart diseases) in the controls. Out of those with anomaly birth history, none of the cases and 85.7% of the controls had preconception folic acid supplementation, which is a statistically significant difference (*p* value; 0.033) [Table 2].

### 3.3. Periconceptional Characteristics of the Study Participants

Out of the total participants, 40.5% (24.3% cases and 16.2% controls) of the pregnancies were unplanned (*p* value; 0.075). One-third (30.6%) of the participants (9.9% cases and 20.7% controls) had preconception care, which is significantly different between cases and controls (*p* value; 0.014). However, only 14.4% of the participants (5.4% cases and 9% controls) had preconception folic acid or multivitamin supplementation (*p* value; 0.364). There was a significant difference (*p* value; 0.001) between cases (3.5%) and controls (30.6%) for use of folic acid or multivitamin in the first three months after conception [Table 3].

### 3.4. Prevalence of Neural Tube Defects

During the study period, there were a total of 8,677 births after the 28th week of gestation. A total of 115 pregnancies were terminated between the 12th and 28th weeks of gestation; of these, 56 pregnancies were terminated because of presence of NTDs. A total of 55 cases of NTDs were documented after the 28th week of gestation. The overall prevalence of NTDs after 12 weeks of gestation was found to be 126 per 10,000 births (95% CI, 100–150). Among live births and stillbirths, excluding medically terminated cases, the birth prevalence of NTDs was 63.4 per 10,000 births (LBs and SBs) (95% CI, 51–77).

**Figure 1:**
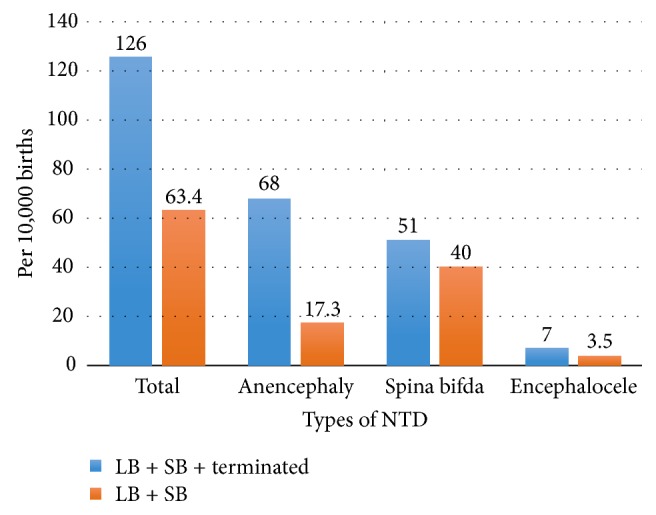
Total and birth prevalence of NTDs, Addis Ababa University Teaching Hospitals, Ethiopia, February 1 to August 30, 2016 (*n* = 333).

Anencephaly (68 cases per 10,000 births), spina bifida (51 cases per 10,000 births), and encephalocele (seven cases per 10,000 births) were the most common types of the NTDs. Excluding terminated cases as a prevention, the birth prevalence of anencephaly was 17.3 cases per 10,000 live and stillbirths; prevalence of encephalocele was 3.5 cases per 10,000 live and stillbirths; and spina bifida prevalence was 40 cases per 10,000 live and stillbirths [Figure 1].

### 3.5. Characteristics of Neural Tube Defects

Out of the total cases of NTDs, 54.1% were cases of anencephaly; 40.5%, spina bifida; and 5.4%, encephalocele. Diagnosis of NTDs was made by ultrasound before birth for 96/111 (86.5%) cases, while the remaining cases were diagnosed at birth even though there was at least one ultrasound scan during ANC. The mean gestational age at diagnosis was 25 weeks (±8) and the mean gestational age at delivery or termination was 29 weeks (±9) [Table 4].

### 3.6. Risk Factors Related to Neural Tube Defects

Family annual cash income, drug use in the first three months of conception, sex of the baby, prepregnancy BMI, and whether the pregnancy was planned or not were independent predictors of neural tube defects. Women who used either folic acid or multivitamins containing folic acid during the first three months of conception were 53% less likely (aOR, 0.47; 95%, 0.23, 0.95) to have NTDs than those who did not take any supplements. Though not statistically significant, women who used any other drugs than folic acid or multivitamins were two times (aOR, 2.12; 95%, 0.98, 4.56) more likely to have NTDs.

Women who had planned pregnancies were 53% less likely (aOR, 0.47; 95%, 0.24, 0.92) to have NTDs as compared to unplanned pregnancies. Male sex was associated with a 44% reduction in the risk of NTDs as compared to female sex (aOR, 0.56; 95%, 0.33, 0.94); however, we have not seen differences between male versus females for anencephaly and spina bifida (anencephaly, 53.8% versus 54.2%; encephalocele, 2.0% versus 8.5%; and spina bifida, 32.7% versus 34.0%, resp.). Women who had a normal or an underweight prepregnancy BMI were 51% less likely to have NTDs than those who were overweight or obese (aOR, 0.49; 95%, 0.29, 0.93). Women with annual family cash income of less than $1,300 USD (aOR, 2.50; 95%, 1.21, 5.52), $1,300–1,800 USD (aOR, 2.82; 95%, 1.33, 5.81), and $1,801–2,700 USD (aOR, 2.64; 95%, 1.21, 5.82) were almost 2.5 times more likely to have NTDs, compared to those with annual cash incomes of greater than $2,700 USD [Table 5].

## 4. Discussion

Because birth defects are a major cause of mortalities before five years of age, adequate surveillance data are needed to develop plausible prevention strategies. This is particularly important for birth defects that can be prevented via well-established interventions, like NTDs [1–3]. Although many births in Ethiopia occur outside of health institution [13], institution-based prevalence studies give some insight into the prevalence and risk factors of NTDs. Such evidence might be a starting point for countries with limited resources like Ethiopia to start a hospital-based NTDs surveillance program that may then develop to a population-based surveillance [16].

In this study of more than 8,000 births, the total prevalence for all types of NTDs was found to be 126 per 10,000 births, and the birth prevalence for all types of NTDs was found to be 63.4 per 10,000 births. This finding is one of the highest NTDs prevalence reported in Africa. It is higher than reports from Yaoundé, Cameroon (18.6 per 10,000) [17]; Democratic Republic of Congo (10.2 per 10,000) [18]; Blantyre, Malawi (6.3 per 10,000) [19]; Cross River and Akwa Ibom states of Nigeria (5.2 per 10,000) [20]; South Africa (9.8 per 10,000) [21]; and Dares Salaam, Tanzania (30.2 per 10,000) [22]. It is higher than most of the Eastern Mediterranean regions including Egypt (16 per 10,000) [23]; Southwest Iran (38.1 per 10,000) [24]; Western Iraq (27.9 per 10,000) [25]; Sudan (33.7 per 10,000) [26]; and the Al-Jahra region of Kuwait (6.5 per 10,000) [27]. It is also higher than all European countries (EUROCAT) [28, 29] and many Southeast Asian regions including Bangladesh (13.8 per 10,000) [30]; Eastern India (17.8 per 10,000) [31]; Nepal (11.9 per 10,000) [32]; and Bangkok, Thailand (6.3 per 10,000) [33]. Reports of similar prevalence to our study have emerged from Northern China (199.4 per 10,000 births) [34]; Eastern Saudi Arabia (53.5 per 10,000) [35]; Jordan (62.9 per 10,000) [36]; Iran (82.9 per 10,000) [37]; and Algeria (75.4 per 10,000) [38]. Consistently high prevalence of NTDs similar to our study has also been reported in Swat (124 per 10,000) [39] and Abbottabad (68.8 per 10,000) [40] regions of Pakistan.

These variations might be explained by the influence of racial, geographical, nutritional, socioeconomic, and biological differences. Rates may also be affected by the differences in national NTDs prevention intervention programs in various countries [41]. Settings and designs could also affect findings from different studies. Referral hospitals would be expected to have higher rates than population-based studies, as many cases are referred to these hospitals for termination and diagnosis of NTDs. Methods of case ascertainment, gestational age at prenatal diagnosis, and infant ages at diagnosis as well as inclusion/exclusion of electively medically terminated cases of NTDs could also vary.

Findings of the current study should be interpreted with caution for the following reasons. First, hospital-based studies may overestimate prevalence of NTDs. Second, our study included medically terminated cases of NTDs, while most other studies report on live births and stillbirths only. Conversely, the high NTDs rate we found in our study could also be the actual prevalence for the study area given similar findings from another retrospective study in the same area [12]. This high prevalence could result from nutritional factors, genetics, lack of routine folic acid supplementation, and absence of folic acid fortification programs. In our study, half of the women started ANC after three months of gestation and only 25% of women had folic acid supplementation in the first 12 weeks of gestation, which is the critical period of supplementation [42].

Our study highlighted the importance of folic acid and/or multivitamin supplementation during the first three months of conception for prevention of NTDs. This finding is in line with other studies worldwide [43, 44] and international recommendations of folic acid supplement for women from the moment they attempt to conceive until 12 weeks of pregnancy [45]. Despite the WHO periconceptional folic acid supplementation recommendations, studies showed that many women still do not follow the recommendations, particularly women of low socioeconomic status [46]. This is also true in our study; though 17% of the participants had preconception care, only 7.8% were supplemented [Table 3].

The association between prepregnancy BMI and occurrence of NTDs was similarly reported elsewhere [47, 48]. This may be because NTD-protective effect of folic acid supplementation is weaker in overweight/obese mothers [49] and obese women may require higher doses of folic acid supplementation to achieve similar serum levels [50]. Regarding the nonmodifiable factors, though the contribution of sex to NTDs is controversial and no plausible biologic explanation is known, other studies report similar findings [51, 52]. Higher rates of NTDs have been reported in populations with lower socioeconomic status from Europe and the USA [53]. Our study found similar associations even though we did not measure economic status based on wealth quantile [13], possibly due to factors like housing condition, frequency of infections, and deficiency of dietary factors such as the intake of vitamins and folic acid.

## 5. Conclusion

The prevalence of neural tube defects at three hospitals in Addis Ababa was found to be higher than other reports from Africa, Europe, America, and many Asian countries. Our finding is consistent with previous findings. Comprehensive preventive strategies focused on the identified risk factors should be urgently established. Moreover, studies on preventive strategies including folic acid supplementation should be conducted in these settings.

## Figures and Tables

**Table 1:** Sociodemographic characteristics of the study participants, Addis Ababa University Teaching Hospitals, Ethiopia, February 1 to August 30, 2016 (*n* = 333).

Variables	Case (%)	Controls (%)	*p* value
Residence			0.867
Addis Ababa	105 (94.6)	209 (94.1)	
Outside Addis Ababa	6 (5.4)	13 (5.9)	
Age of participants (mean; SD)	26.9; ±4.6	26.7; ±5.2	0.193
≤20	12 (10.8)	18 (8.1)	
21–25	44 (39.6)	74 (33.3)	
26–30	35 (31.5)	84 (37.8)	
31–35	12 (10.8)	38 (17.1)	
>35	8 (7.2)	8 (3.7)	
Age of partners (mean; SD)	32.0; ±7	32.5; ±7	0.252
20–29	42 (37.8)	82 (36.9)	
30–39	57 (51.4)	101 (45.5)	
≥40	12 (10.8)	39 (17.6)	
Religion			0.929
Christian	83 (74.8)	165 (74.3)	
Muslim	28 (25.2)	7 (25.7)	
Marital status			0.339
Married	106 (95.5)	206 (92.8)	
Others	5 (4.5)	16 (7.2)	
Educational status			0.532
No formal education	14 (12.6)	23 (10.4)	
Primary school	46 (41.4)	78 (35.1)	
Secondary school	28 (25.2)	68 (30.6)	
Above secondary	3 (20.7)	53 (23.9)	
Partner education status			0.269
No formal education	10 (9)	20 (9)	
Primary school	37 (33.3)	52 (23.4)	
Secondary school	30 (27)	67 (30.2)	
Above secondary	34 (30.6)	83 (37.4)	
Occupation			0.104
Employed	31 (27.9)	62 (27.9)	
Merchant	10 (9)	43 (19.4)	
Daily laborer	12 (10.8)	29 (13.1)	
Housewife	58 (52.3)	88 (39.6)	
Family size (mean; SD)	3.0; ±1.2	3.2; ±1.3	0.061
≤3	81 (73)	139 (62.6)	
>3	30 (27)	83 (37.4)	
Family annual cash income (USD)	889; ±964	2310; ±1605	0.032
(mean; SD)			
$<1300	29 (26.1)	49 (22.1)	
$1300–1800	35 (31.5)	55 (24.8)	
$1801–2700	27 (24.3)	43 (19.4)	
$>2700	20 (18)	75 (33.8)	
Prepregnancy BMI (mean; SD)	23; ±3.1	22; ±2.8	0.041
<24.9	85 (76.6)	190 (85.6)	
≥25	26 (23.4)	32 (14.4)	

BMI: body mass index; SD: standard deviation; USD: United States Dollar.

**Table 2:** Obstetric histories of the study participants, Addis Ababa University Teaching Hospitals, Ethiopia, February 1 to August 30, 2016 (*n* = 333).

Variables	Case (%)	Control (%)	*p* value
Previous history of abortion			0.198
Yes	21 (18.9)	56 (25.2)	
No	90 (81.1)	166 (74.8)	
Parity (mean ± SD)	1.2 (±1.1)	1.9 (±1.1)	0.289
Nulliparous	33 (29.7)	0 (0)	
Prim parous	45 (40.5)	105 (47.3)	
Multiparous	33 (29.7)	117 (52.7)	
END^*∗*^ history			**0.042**
Yes	7 (6.3)	4 (1.8)	
No	104 (93.7)	218 (98.2)	
Stillbirth history			0.364
Yes	2 (1.8)	8 (3.6)	
No	109 (98.2)	214 (96.4)	
Congenital anomaly birth History			0.820
Yes	3 (2.7%)	7 (3.2)	
No	108 (97.3)	215 (96.8)	
Types of congenital anomaly (*n* = 10)			
Cardiac	0 (0)	2 (28.6)	
NTDs^*∗∗∗*^	3 (100)	5 (71.4)	
Preconception folic acid supplementation (*n* = 10)			**0.033**
Yes	0 (0)	6 (85.7)	
No	3 (100)	1 (14.3)	
Number of index Pregnancy			0.334
Singleton	108 (97.3)	211 (95)	
Twin	3 (2.7)	11 (5)	
Sex			0.074
Male	52 (46.8)	127 (57.2)	
Female	59 (53.2)	95 (42.8)	
ANC^*∗∗*^ initiation time			**0.003**
1st trimester	46 (41.4)	127 (57.2)	
2nd trimester	63 (56.8)	83 (37.4)	
3rd trimester	2 (1.8)	12 (5.4)	
Place of ANC			0.014
Private	10 (9)	17 (7.7)	
clinic/hospital			
Health center	93 (83.8)	158 (71.2)	
Public hospitals	7 (6.3)	44 (19.8)	
No ANC	1(.9)	3 (1.4)	

^*∗*^Early neonatal death. ^*∗∗*^Antenatal care. ^*∗∗∗*^Neural tube defects.

**Table 3:** Periconceptional characteristics of the study participants, Addis Ababa University Teaching Hospitals, Ethiopia, February 1 to August 30, 2016 (*n* = 333).

Variables	Cases (%)	Controls (%)	*p* value
Any chronic illness before conception			0.104
Yes	6 (5.4)	24 (10.8)	
No	105 (94.6)	198 (89.2)	
Current pregnancy type			0.075
Planned	84 (75.7)	186 (83.8)	
Unplanned	27 (24.3)	36 (16.2)	
Remember current LNMP^*∗*^			0.108
Yes	77 (69.4)	134 (60.4)	
No	34 (30.6)	88 (39.6)	
Contraceptive use before conception			0.072
Yes	55 (49.5)	133 (59.9)	
No	56 (50.5)	89 (40.1)	
Preconception care			**0.014**
Yes	11 (9.9)	46 (20.7)	
No	100 (90.1)	176 (79.3)	
Preconception drugs use			0.364
Folic acid/multivitamins	6 (5.4)	20 (9)	
Any other drugs	3 (2.7)	3 (1.4)	
None	102 (91.9)	199 (89.6)	
Any attempt to terminate the pregnancy			0.076
Yes	5 (4.5)	3 (1.4)	
No	106 (95.5)	219 (98.6)	
Drugs use in the 1st trimester			**0.001**
Folic acid/multivitamins	15 (13.5)	68 (30.6)	
Any other drugs	22 (19.8)	23 (10.4)	
None	74 (66.7)	131 (59.0)	

^*∗*^Last normal menstrual period.

**Table 4:** Characteristics of neural tube defects, Addis Ababa University teaching hospitals, Ethiopia, February 1 to August 30, 2016 (*n* = 111).

Variables	Frequency	Percentage	Remark
Types of NTDs			
Anencephaly	60	54.1	
Spina bifida	45	40.5	
Encephalocele	6	5.4	
Time at diagnosis			
Antenatal	96	86.5	
At birth	15	13.5	
US^*∗*^ scan done for at birth diagnosis			*n* = 15
Yes	13	86.7	
No	2	13.3	
Gestational age at Diagnosis (weeks) (*n* = 96)			Mean = 25 (±8)
<24	47	42.3	
24–27^6/7^	18	16.2	
28–33^6/7^	12	10.8	
34–36^6/7^	8	7.2	
≥37	11	9.9	
Gestational age at Delivery/expulsion (weeks)			Mean = 29 (±9) Median = 27.7
<24	35	31.5	
24–27^6/7^	21	18.9	
28–33^6/7^	15	13.5	
34–36^6/7^	6	5.4	
≥37	34	30.6	
Mode of delivery/termination			
Spontaneous vaginal delivery	38	34.2	
Induced vaginal delivery	58	52.3	
Cesarean section	8	7.2	
Destructive vaginal delivery	7	6.3	
Admission to delivery/expulsion in days			Mean = 3.2 (±2.2) Median = 3
1 day	40	36.0	
2–4 days	44	39.6	
≥5 days	27	24.3	

^*∗*^Ultrasound.

**Table 5:** Logistic regression analysis on determinants of neural tube defects, Addis Ababa University Teaching Hospitals, Ethiopia, 2016 (*n* = 333).

Variables	Neural tube defects		*p* value	OR (95% CI)	
	Yes	No		COR	AOR
*Annual family cash income (USD)*			0.032		
<$1300	29	49		2.2 (1.13–4.35)	**2.50 (1.21–5.52)**
$1300–1800	35	55		2.39 (1.25–4.57)	**2.82 (1.33–5.81)**
$1801–2700	27	43		2.36 (1.18–4.69)	**2.64 (1.21–5.82)**
>$2700	20	75		1	1
*Drugs in the 1st trimester*			**0.001**		
Folic acid/multivitamins	15	68		0.39 (0.28–0.73)	**0.47 (0.23–0.95)**
Any other drugs	22	23		1.69 (0.88–3.25)	2.12 (0.98–4.56)
None	74	131		1	1
*Prepregnancy BMI*			0.043		
<24.9	85	190		0.55 (0.31–0.98)	**0.49 (0.29–0.95)**
≥25	26	32		1	1
*Place of ANC*			0.022		
Private clinic/hospital	10	17		1.77 (0.16–19.3)	8.34 (0.57–121.3)
Health center	93	158		1.77 (0.18–17.2)	4.76 (0.41–55.67)
Public hospitals	7	44		0.48 (0.04–5.26)	1.68 (0.12–22.75)
No ANC	1	3		1	1
*END history*			0.042		
Yes	7	4		3.67 (1.05–12.80)	4.15 (0.95–18.16)
No	104	218		1	1
*Contraceptive use before conception*					
Yes	55	133		0.66 (0.42–1.04)	0.67 (0.40–1.12)
No	56	89		1	1
*Outcome (sex)*			0.075		
Male	52	127		0.66 (0.42–1.04)	**0.56 (0.33–0.94)**
Female	59	95		1	1
*Pregnancy type*			0.077		
Planned	84	186		0.60 (0.34–1.06)	**0.47 (0.24–0.92)**
Unplanned	27	36		1	1
*Remember LNMP*			0.109		
*Yes*	77	134		1.49 (0.92–2.41)	1.96 (0.98–3.40)
*No*	34	88		1	1
*Preconception care*			0.016		
Yes	11	46		0.42 (0.21–0.85)	0.50 (0.21–1.21)
No	100	176		1	1

END: early neonatal death; BMI: body mass index; ANC: antenatal care; LNMP: last normal menstrual period.

## Additional Points

*Strength/Limitations of This Study*. This study has several strengths. First, it included data from evaluation of more than 8,000 births and cases of terminated pregnancy. Second, it employed the principles of active surveillance of case detection so that the risk of missed cases of NTDs was minimized. Finally, analyses were based on all birth outcomes (stillbirths, live births, and terminated pregnancy). Data were also collected in the immediate postpartum period to minimize the recall bias of the periconceptional event.

Although not unique to this study, a potential limitation is participant recall and self-report of risk factors, which could introduce bias. The self-reported nature of the information income and other related factors are also a limitation of this study. It is a hospital-based study and could have missed many deliveries which occurred outside hospitals.
